# Supplementary material for: Resolving the full spectrum of human genome variation using Linked-Reads
Source: Genome Res. 2019 Apr;29(4):635–45. doi: 10.1101/gr.234443.118 (PMC6442396; doi:10.1101/gr.234443.118)
Supplement: Supplemental Material [file supp_gr.234443.118_Supplemental_longranger-2.2.zip › longranger-2.2.tar.bz2!/lr2.2-public/lib/stan/cmdstan/stan/doc/api/html/print__progress_8hpp_source.html]

Stan: src/stan/common/print\_progress.hpp Source File


|  |  |
| --- | --- |
|  | Stan  2.5.0  probability, sampling & optimization |


- Main Page
- Related Pages
- Namespaces
- Classes
- Files

- File List
- File Members

All Classes Namespaces Files Functions Variables Typedefs Enumerations Enumerator Friends Macros Pages

- src
- stan
- common

print\_progress.hpp

Go to the documentation of this file.

1 #ifndef STAN\_\_COMMON\_\_PRINT\_PROGRESS\_HPP

2 #define STAN\_\_COMMON\_\_PRINT\_PROGRESS\_HPP

3

4 #include <cmath>

5 #include <iomanip>

6 #include <stan/common/do\_print.hpp>

7

8 #include <iostream>

9

10 namespace stan {

11  namespace common {

12

13  void print\_progress(const int m,

14  const int start,

15  const int finish,

16  const int refresh,

17  const bool warmup,

18  const std::string prefix,

19  const std::string suffix,

20  std::ostream& o) {

21  int it\_print\_width = std::ceil(std::log10((double) finish));

22  if (do\_print(m, (start + m + 1 == finish), refresh)) {

23  o << prefix;

24  o << "Iteration: ";

25  o << std::setw(it\_print\_width) << m + 1 + start

26  << " / " << finish;

27  o << " [" << std::setw(3)

28  << static\_cast<int>( (100.0 \* (start + m + 1)) / finish )

29  << "%] ";

30  o << (warmup ? " (Warmup)" : " (Sampling)");

31  o << suffix;

32  o << std::flush;

33  }

34  }

35

36  } // namespace common

37

38 } // namespace stan

39

40 #endif

stan::common::do\_print

bool do\_print(const int n, const bool special, const int refresh)

Indicates whether it should print on the current iteration.

**Definition:** do\_print.hpp:25

stan::agrad::ceil

fvar< T > ceil(const fvar< T > &x)

**Definition:** ceil.hpp:15

do\_print.hpp

stan::common::warmup

void warmup(stan::mcmc::base\_mcmc \*sampler, int num\_warmup, int num\_samples, int num\_thin, int refresh, bool save, stan::io::mcmc\_writer< Model, SampleRecorder, DiagnosticRecorder, MessageRecorder > &writer, stan::mcmc::sample &init\_s, Model &model, RNG &base\_rng, const std::string &prefix, const std::string &suffix, std::ostream &o, StartTransitionCallback &callback)

**Definition:** warmup.hpp:13

stan::common::print\_progress

void print\_progress(const int m, const int start, const int finish, const int refresh, const bool warmup, const std::string prefix, const std::string suffix, std::ostream &o)

**Definition:** print\_progress.hpp:13

stan::agrad::log10

fvar< T > log10(const fvar< T > &x)

**Definition:** log10.hpp:15


---

[ Stan Home Page ]

*© 2011–2014,
Stan Development Team.*
